# Asparaginyl endopeptidase contributes to cetuximab resistance via MEK/ERK signaling in RAS wide-type metastatic colorectal cancer

**DOI:** 10.1007/s12094-022-02986-6

**Published:** 2023-01-07

**Authors:** Xiaojing Xu, Mengling Liu, Ke Peng, Yiyi Yu, Tianshu Liu

**Affiliations:** 1 Department of Oncology, Zhongshan Hospital, Fudan University, Shanghai, 200032 China; 2 Cancer Center, Zhongshan Hospital, Fudan University, Shanghai, 200032 China; 3 Center of Evidence-Based Medicine, Fudan University, 180 Feng Lin Road, Shanghai, 200032 China

**Keywords:** Metastatic colorectal cancer, Cetuximab, Resistance, Asparaginase endopeptidase

## Abstract

**Background:**

Cetuximab, a monoclonal antibody targeting epidermal growth factor receptor (EGFR), is effective for *RAS* wild-type metastatic colorectal cancer (mCRC) patients. However, cetuximab resistance often occur and the mechanism has not been fully elucidated. The purpose of this study was to investigate the role of asparaginyl endopeptidase (AEP) in cetuximab resistance.

**Methods:**

Differentially expressed genes between cetuximab responders and non-responders were identified by analyzing the gene expression profile GSE5851, retrieved from Gene Expression Omnibus (GEO). The potential genes were further validated in cetuximab-resistant CRC cell lines. The expression of AEP in the peripheral blood and tumor tissues of mCRC patients in our hospital were detected by enzyme-linked immunosorbent assay (ELISA) and immunohistochemistry, respectively. The survival analysis was carried out by Kaplan–Meier method. The function and associated pathways of AEP were further investigated by lentivirus transfection, CCK8 assay, colony formation assay, real-time polymerase chain reaction (qPCR) and western blot.

**Results:**

Through bioinformatics analysis, we found that the expression of AEP gene was related to progress free survival (PFS) of mCRC patients treated with cetuximab alone (*P* = 0.00133). The expression of AEP was significantly higher in the cetuximab-resistant CRC cell lines, as well as in mCRC patients with shorter PFS treated with cetuximab-containing therapy. Furthermore, AEP could decrease the sensitivity of CRC cells to cetuximab in vitro. And the phosphorylation level of MEK and ERK1/2 was increased in AEP overexpression cells. The downregulation of AEP using specific inhibitors could partially restore the sensitivity of CRC cells to cetuximab.

**Conclusion:**

The higher expression of AEP could contribute to the shorter PFS of cetuximab treatment in mCRC. The reason might be that AEP could promote the phosphorylation of MEK/ERK protein in the downstream signal pathway of EGFR.

**Supplementary Information:**

The online version contains supplementary material available at 10.1007/s12094-022-02986-6.

## Introduction

Colorectal cancer (CRC) is one of the most common malignant tumors worldwide [1]. Among them, 40–50% patients had metastasis at the time of initial diagnosis, so the treatment of metastatic CRC (mCRC) is particularly crucial. Over the past decade, monoclonal antibodies targeting epidermal growth factor receptor (EGFR), such as cetuximab and panitumumab, have obviously improved overall survival and disease control in mCRC [2–4]. The combinations of cetuximab and chemotherapeutic regimens, such as FOLFIRI, FOLFOX or FOLFOXIRI, have been the most important strategies for the treatment of *RAS* wild-type mCRC [2, 5, 6]. However, resistance often occur in less than nine months after cetuximab treatment. A number of genetic mutations were found to contribute to the resistance [7–9], including mutation of *RAS* [10–13], *BRAF* [12, 14], *PIK3CA* [12, 15] and amplification of *HER2* [16] and *MET* [17]. Despite these biomarkers, additional mechanisms of resistance to EGFR blockade are thought to exist in mCRC.

Asparaginyl endopeptidase (AEP), also known as legumain (*LGMN*), is a member of the C13 cysteine protease family [18]. The inactive precursor of AEP can be self-activated in acidic environment to become an active mature enzyme, which mainly exists in the introsome or lysosome. In recent years, it is reported that AEP is also expressed in cytoplasm and nucleus [19, 20]. Many studies have shown that the expression of AEP increased in various tumors [21], such as breast cancer [22], ovarian cancer [23], colorectal cancer [20], gastric cancer [24, 25] and glioblastoma [26]. Furthermore, the higher level of AEP indicated poorer prognosis and shorter survival in these patients. However, the mechanism has not been elucidated.

Through the Gene Expression Omnibus (GEO) database, using the baseline gene expression profile (GSE5851) of *KRAS* wild-type mCRC patients before cetuximab treatment, we found that the level of AEP was closely related to the progression free survival (PFS) (*P* = 0.00133). Patients with higher AEP had shorter PFS. The purpose of current study was to further validate the role of AEP in cetuximab resistance in mCRC.

## Methods

### Microarray profile analysis from gene expression omnibus (GEO) database

The gene expression profile GSE5851 was downloaded from the GEO database. GSE5851 contained 80 *KRAS* wide-type mCRC patients who were enrolled for treatment with cetuximab monotherapy [27]. The microarrays were conducted by Affymetrix GeneChip Scanner 3000 with GPL571 using pre-treatment tumor tissue samples. Statistical analyses were performed using quantile normalized signal intensity values. Univariate analysis to compare gene expression profiles according to clinical response was performed using a two-sided unequal variance *t* test. *P* value < 0.05 was considered significant.

### Patients

Forty-four mCRC patients treated in our department from August 2016 to April 2017 and provided informed consent for their biological samples to be use in future research. The patients were all histologically diagnosed with advanced or metastatic *RAS* wide-type colorectal adenocarcinoma with no previous palliative therapy. All of them received first-line treatment with chemotherapy plus cetuximab. Tumor tissue samples were collected before treatment. The study was approved by Ethics Committee of Zhongshan Hospital Affiliated to Fudan University.

### Cell culture

Caco2 and NCI-H508 cell line was obtained from Cell Bank of the Chinese Academy of Sciences (Shanghai, China). Caco2 cells were cultured in Dulbecco’s Modified Eagle’s Medium (Gibco, USA) containing 10% FBS (Gibco, USA) and 1% penicillin/streptomycin (Invitrogen, USA). NCI-H508 cells were maintained in RMPI-1640 medium (Gibco, USA) supplemented with 10% FBS and 1% penicillin/streptomycin in a 5% CO_2_ humidified atmosphere at 37 °C. In order to verify the role of AEP or MEK/ERK pathways, AEP inhibitors (AEPI, obtained from Lin [26]) or MEK inhibitors U0126 (Cell Signaling Technology, 9903) were used for cell intervention.

### Cetuximab-resistant CRC cells establishment

NCI-H508 cells, known as cetuximab-sensitive CRC cells, were continuously exposed to increasing concentrations of cetuximab for 6 months. The IC50 value for cetuximab of NCI-H508 cells was about 1 μg/mL. The drug dose was progressively increased to 10 μg/mL in approximately 2 months, to 50 μg/mL after other 2 months, and finally, to 100 μg/mL after additional 2 months. The established cetuximab-resistant NCI-H508 cell line (NCI-H508-CR) was then maintained in continuous culture with this maximally achieved dose of cetuximab that allowed cellular proliferation.

### Cell proliferation assay

For cell proliferation assay, the viability of CRC cell lines was determined by Cell Counting Kit 8 (CCK8) (Dojindo, Japan) and measured at OD450 nm with the BioTek Gen5 system (BioTeck, USA). For clonogenic assay, cells were plated in 6-well plates with 2 ml media. Media was changed every 3 to 4 days. After 7–10 days, colonies were fixed in 80% methanol and stained with 0.1% crystal violet.

### Real-time polymerase chain reaction (qPCR)

RNA isolation was performed using the TRIZOL reagent (Invitrogen, USA). The cDNA was prepared using an oligo (dT) primer (Supplement Table 1) and reverse transcriptase (Takara, Shiga, Japan) following standard protocols. Quantitative real-time polymerase chain reaction (qRT-PCR) was performed using SYBR Green on the ABI 7500 real-time PCR System (Applied Biosystems, Foster City, CA). Relative expression was presented using the 2^−ΔCt^ method [ΔCt = Ct (chemokine) − Ct (beta-actin)].

### Western blot

Cell samples were collected and lysed in RIPA buffer with protease inhibitor cocktails. Total cell protein extracts (20 µg/lane) were subjected to SDS-PAGE analysis. The membrane was blocked with 5% milk in TBST before incubation in the following antibody overnight at 4 °C, AEP antibody (R&D Systems, AF2199) or EGFR antibody (Cell Signaling Technology, 2085), phosphor-EGFR antibody (Cell Signaling Technology, 3777), MEK1/2 (Cell Signaling Technology, 4694), phosphor-MEK1/2 (Cell Signaling Technology, 9154), ERK1/2 (Cell Signaling Technology, 4695), phosphor-ERK1/2 (Cell Signaling Technology, 4370), beta-actin (Cell Signaling Technology, 3700). The membranes were washed with TBST and incubated with the secondary antibodies (Santa Cruz Biotechnology). The immunoreactive proteins were visualized by chemiluminescence reagents (ECL; Amersham Biosciences).

### ELISA

Levels of AEP protein in the cell supernatant were determined using ELISA kit (R&D Systems, DY4769) in accordance with the protocol provided by the manufacturer. Briefly, samples and standards were added in a 96-well polystyrene microplate coated with diluted AEP capture antibody and incubated for 2 h. The plates were washed, added with AEP detection antibody, and incubated for 2 h. The working dilution was added after washing twice. Cover the plate and incubate for 20 min at room temperature. Substrate solution was added to each well and incubate for 20 min at room temperature. The reaction was terminated with stop solution. Then, determine the optical density of each well immediately, using a microplate reader set to 450 nm.

### Immunohistochemistry

Slides were routinely de-paraffinizated and rehydrated, and then heated at 98 °C in a citrate buffer for 20 min and cooled naturally to room temperature. Sections were incubated in 0.3% hydrogen peroxide for 20 min and blocked with 5% normal horse serum in PBS for 30 min. AEP antibody (R&D Systems, AF2199) was added for incubating overnight at 4 °C, then stained using a highly sensitive streptavidin–biotin–peroxidase detection system and counterstained with hematoxylin.

### Lentiviral vector transfection

The target sequences for AEP shRNAs were 5′-gatccGATGGTGTTCTACATTG-AATTCAAGAGATTCAATGTAGAACACCATCTTTTTTg-3′ (AEP-KD1) and 5′-gatccAAACTGATGAACACCAATGATTTCAAGAGAATCATTGGTGTTCATCAGTTTTTTTTTg-3′ (AEP-KD2). After 48 h, the efficiency of AEP knockdown was confirmed via western blot and ELISA. Lentiviral vectors for human AEP-shRNA carrying a green fluorescent protein (GFP) sequence were constructed by Hanyin Co. (Shanghai, China). To obtain the stable AEP knockdown cell line, cells were seeded in six-well dishes at a density of 2 × 10^5^ cells per well. The cells were then infected with the same titer virus with 8 μg/ml polybrene on the following day. Approximately 72 h after viral infection, GFP expression was confirmed under a fluorescence microscope, and the culture medium was replaced with selection medium containing 4 μg/ml puromycin. The cells were then cultured for at least 14 days. The puromycin-resistant cell clones were isolated, amplified in medium containing 2 μg/ml puromycin for seven to nine days, and transferred to a medium without puromycin. The clones were designated as AEP-KD or NC cells.

### Colony formation experiment

Cells were seeded in six-well dishes at a density of 1000 cells per well and cultured at 37 °C, 5% CO_2_. When clones were visible, the culture was stopped. The supernatant was removed by vacuum pump. 80% methanol was added at room temperature for 20 min. Crystal violet staining solution was added dropwise, and the colonies were counted by software image J.

### Immunofluorescence assay

Cells fixed on coverslips were treated with the AEP antibody (R&D Systems, AF2199) and secondary antibodies conjugated with Alexa Fluor-488 (Abcam). Then, coverslips were washed with PBS, stained with 4′, 6-diamidino-2-phenylindole (DAPI, Yeasen, China), and evaluated using laser confocal microscopy (Leica TCS SP5 II, Wetzlar, Germany).

### Statistical analysis

The median PFS was calculated by Kaplan–Meier survival analysis, and its significance was evaluated by log rank test. The data results of continuous variables were expressed by mean ± standard deviation, and the difference between the two groups was analyzed by *t* test. *P* value of < 0.05 was considered statistically significant. SPSS 24 software was used for statistical analysis.

## Results

### Identification of differentially expressed genes (DEGs)

**Fig. 1:**
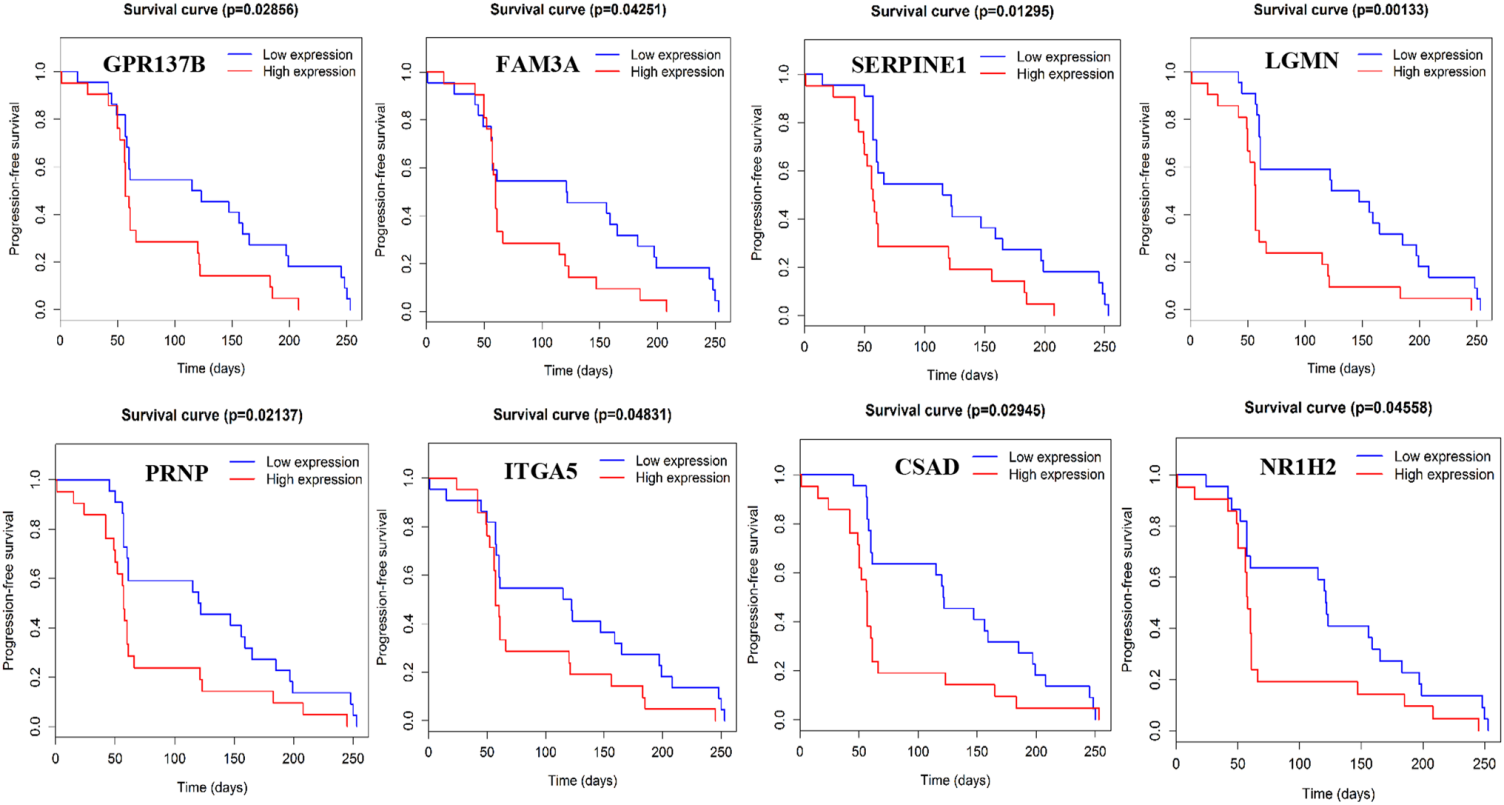
Identification of differentially expressed genes. Eight candidate genes upregulated in cetuximab non-responders. The expression of asparaginyl endopeptidase gene (LGMN) was significantly related to the progression free survival (PFS) (*P* = 0.00133)

A total of 1002 DEGs were identified from GSE5851 data set. Among them, 343 genes were upregulated in non-responders of cetuximab in mCRC. The top 100 upregulated genes are shown in Supplement Table 2. By searching previous literature, eight candidate genes (Fig. 1) were identified which were closely related to tumor development. Among them, the expression of asparaginyl endopeptidase gene (LGMN) was significantly related to the progression free survival (PFS) (*P* = 0.00133) (Fig. 1). Patients with higher LGMN expression had shorter PFS of cetuximab.

### AEP increased in cetuximab-resistant cells

**Fig. 2:**
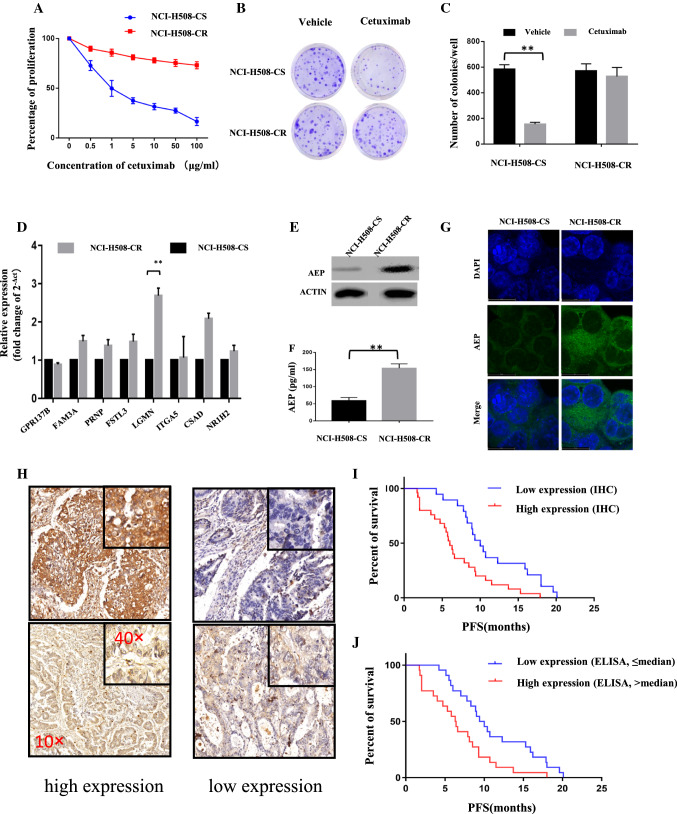
AEP increased in cetuximab-resistant cells and patients. **A** CCK8 assay showed that the proliferation of NCI-H508-CR cells after cetuximab intervention did not decrease. **B**, **C** Number of colonies did not decrease in NCI-H508-CR cells after cetuximab intervention. **D** Eight candidate genes were identified by qPCR in NCI-H508-CR cells (**P < 0.01). **E**, **F** AEP expression results of western blot and ELISA (**P < 0.01). **G** Subcellular localization of AEP was assessed by immunofluorescence staining (100×). **H** Expression level of AEP in tumor tissue detected by immunohistochemistry (10× , 40×). **I**, **J** The median PFS of patients with high expression of AEP was shorter than that of patients with low expression of AEP in tissue (Figure **I**: 6 months vs 10 months, *P* = 0.0023) and in serum (Figure **J**: 6.35 months vs 9.7 months, *P* = 0.0055)

CCK8 assay and colony formation assay were used to verify the function of cetuximab-resistant cells. We found that the proliferation and colony formation of NCI-H508-CR cells after treatment with cetuximab did not decrease, compared with its parallel NCI-H508 cells (Fig. 2A–C). Eight candidate genes were identified by qPCR in these cells. The results showed that the expression of AEP gene in NCI-H508-CR cells was significantly higher than that in NCI-H508 cells (*P* < 0.01), while there was no significant difference in the expression levels of other seven genes (Fig. 2D). Also, the expression of AEP protein in NCI-H508-CR cells was significantly elevated as shown in the results of western blot and ELISA (*P* < 0.01) (Fig. 2E, F). Data of immunofluorescence staining and confocal microscopy indicated that cytoplasmic localization of AEP was increased in NCI-H508-CR cells (Fig. 2G).

### AEP increased in cetuximab-resistant patients

From August 2016 to April 2017, tissue and serum samples from 44 patients with mCRC treated with cetuximab-containing therapy in our department were collected and detected by immunohistochemistry and ELISA, respectively. The baseline characteristics are shown in Table 1. The immunohistochemical scoring was defined as follows: according to the staining intensity, it could be divided into 0 (no staining), 1 (light staining), 2 (moderate staining) and 3 (strong staining); according to the staining area, it could be divided into 1 point (≤ 25%), 2 points (> 25%, ≤ 50%), 3 points (> 50%, ≤ 75%), 4 points (> 75%). Multiply the scores of staining intensity and staining area, and the product was between 0 and 12 points. 0–6 was defined as low expression, and 7–12 was defined as high expression (Fig. 2H). The median level of serum AEP expression was 218.35 pg/ml, ranging from 101.67 to 423.33 pg/ml. Taking the median level as the cut-off, the expression level of AEP was divided into high expression (> 218.35 pg/ml) and low expression (≤ 218.35 pg/ml). The results showed that the expression of AEP in tumor tissues or serum was closely related to PFS (Fig. 2I, J). The median PFS of patients with high expression of AEP was shorter than that of patients with low expression of AEP (tissue: 6 months vs 10 months, *P* = 0.0023; serum: 6.35 months vs 9.7 months, *P* = 0.0055).

**Table 1:** Baseline characteristics of patients

Baseline characteristics	*N* (%) (*N* = 44)
Age, *n* (%)	
≤ 60	22 (50)
> 60	22 (50)
Gender, *n* (%)	
Male	30 (68.2)
Female	14 (31.8)
ECOG PS, *n* (%)	
0	11 (25)
1	33 (75)
Number of metastatic organs	
Single	14 (31.8)
Multiple	30 (68.2)
Treatment	
E + FOLFIRI	26 (59.1)
E + FOLFOX	18 (40.9)

### Knockdown of AEP restore the sensitivity of cetuximab

**Fig. 3:**
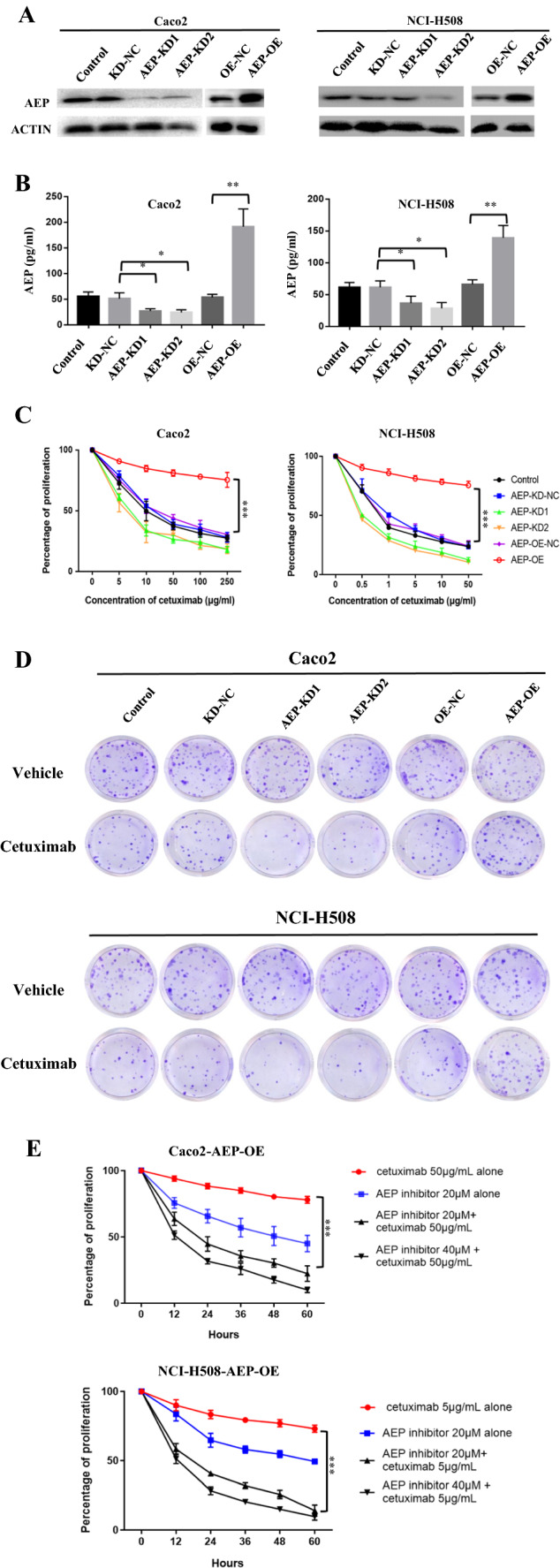
Knockdown of AEP restore the sensitivity of cetuximab. **A** Western blot and **B** ELISA in Caco2 and NCI-H508 cells showed that the expression of AEP in AEP-KD group was significantly decreased (*P < 0.05, **P < 0.01). **C** CCK8 assay showed that the proliferation of AEP-KD cells was significantly decreased than that of the control group (AEP-NC cells) after cetuximab intervention for 48 h (0–250 μg/ml in Caco2 cells and 0–50 μg/ml in NCI-H508 cells) (***P < 0.001). **D** The colony formation of AEP-KD cells was also significantly decreased after cetuximab intervention (Caco2 cell with 50 μg/mL cetuximab intervention for 48 h and NCI-H508 cell with 5 μg/mL cetuximab intervention for 48 h. **E** The proliferation of AEP-OE cells was significantly decreased after AEP inhibitor and cetuximab intervention (***P < 0.001)

Results of western blot and ELISA in Caco2 and NCI-H508 cells showed that the expression of AEP in AEP-KD group was significantly decreased (Fig. 3A, B). Cells was treated with a series of concentration of cetuximab for 48 h (0–250 μg/ml in Caco2 cells and 0–50 μg/ml in NCI-H508 cells). CCK8 assay showed that the proliferation of AEP-KD cells was significantly decreased than that of the control group (AEP-NC cells) after cetuximab intervention (*P* < 0.001) (Fig. 3C), the same as the results of colony formation assay (*P* < 0.001) (Fig. 3D). However, the proliferation and colony formation of AEP-OE cells did not change obviously. The AEP-OE cells were treated with AEP inhibitor (20 μM or 40 μM) for different hours, and the proliferation was significantly decreased after cetuximab intervention (Fig. 3E). The results showed that AEP inhibitor could partially restore the sensitivity to cetuximab in AEP-OE cells.

### Phosphorylation of MEK/ERK in AEP-OE cells

**Fig. 4:**
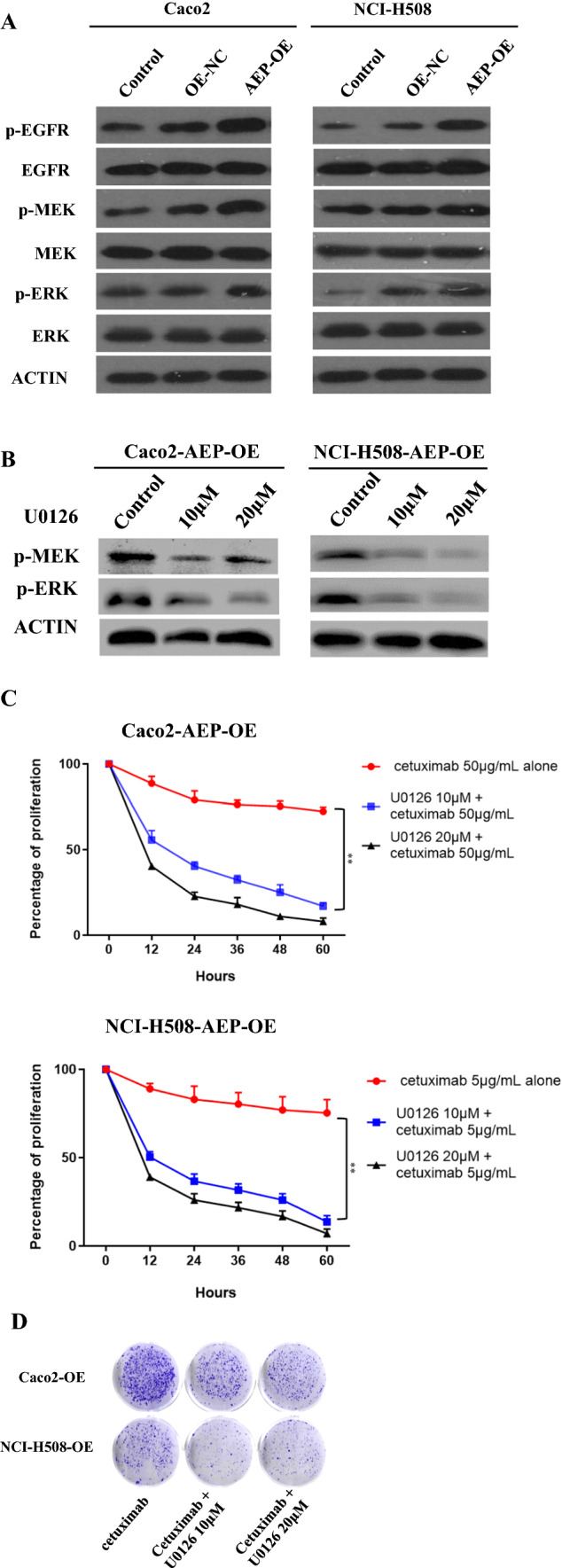
Phosphorylation of MEK/ERK in AEP-OE cells. **A** Western blot showed that the phosphorylation level of MEK and ERK 1/2 increased in AEP-OE cells. **B** ERK inhibitor inhibited the phosphorylation of ERK. **C** The proliferation of AEP-OE cells treated with U0126 was significantly decreased after cetuximab intervention (**P < 0.01). **D** The colony formation of AEP-OE cells was also significantly decreased after cetuximab and U0126 intervention

The results of Western blot showed that the phosphorylation level of MEK and ERK 1/2 increased in AEP-OE cells (Fig. 4A). As shown in Fig. 4B, ERK specific inhibitor, U0126, significantly inhibited the phosphorylation of ERK. The AEP-OE cells were treated with U0126 (10 μM or 20 μM) for different hours, and the proliferation was significantly decreased after cetuximab intervention (Fig. 4C). The results showed that ERK inhibitors could also restore the sensitivity to cetuximab in AEP-OE cells. The colony formation of AEP-OE cells was also significantly decreased after cetuximab and U0126 intervention (Fig. 4D).

## Discussion

In this study, we identified differentially expressed genes between cetuximab responders and non-responders by analyzing the gene expression profile GSE5851, retrieved from Gene Expression Omnibus (GEO). We found that the expression level of AEP was closely related to the PFS of cetuximab treatment.

The GSE5851 profile was reported in 2007 [27], containing 80 *KRAS* wide-type mCRC patients who were enrolled for cetuximab monotherapy. Therefore, it is a very suitable database for analysis of biomarkers for cetuximab. Over the past decade, the combinations of cetuximab and chemotherapeutic regimens have been the standard treatment of *RAS* wild-type mCRC [2, 5, 6], and few patients are treated with cetuximab monotherapy in clinic practice. As a result, cetuximab resistance research have been interfered by the addition with chemotherapeutic drugs. Based on the cetuximab-sensitive cell line NCI-H508, we established its parallel cetuximab-resistant cell line NCI-H508-CR by continuous exposure of cetuximab as described in previous report [28], to further explore the resistant mechanism in vitro.

Since 2003, it has been well established that AEP is widely expressed in various cancers, such as glioblastoma [26], esophageal cancer [29], breast cancer [22], ovarian cancer [23]. High AEP expression was associated with progression and poor prognosis and considered as a potential therapeutic target. Down-regulation of AEP significantly reduced the migration and invasion ability of cancer cells [30]. In colorectal cancer, a study [20] showed that AEP was highly expressed in tumor and stromal cells and could be detected in nucleus in 30% of tumors. Higher AEP expression was associated with shorter overall survival and metastasis free survival. In glioblastoma patients [26], overexpression of AEP promoted tumorigenesis and shortened the survival time. Knock down or pharmacological inhibition of AEP reduced tumorigenesis and prolonged survival in murine models. Our previous study also found that metastatic gastric cancer patients with low expression of AEP achieved more complete response or partial response after chemotherapy [31]. Therefore, AEP may not only be an independent indicator of poor prognosis, but also be involved in the occurrence of drug resistance. The current study focused on the relationship between AEP and cetuximab resistance in colorectal cancer. First, we verified the higher cytoplasmic expression of AEP in cetuximab-resistant cells compared with sensitive cells in vitro. Then, we found that the higher expression of AEP indicated the shorter PFS in the mCRC patients treated with cetuximab-containing therapy.

We further studied the possible mechanisms for AEP-mediated cetuximab resistance. In our previous report, AEP could activate the MAPK/MEK/ERK signaling pathway and promote resistance to microtubule inhibitors in gastric cancers cells, including paclitaxel, docetaxel, and T-DM1 [31]. Cetuximab is an anti-EGFR immunoglobulin G1 (IgG1) mAb. Gene mutations in the EGFR downstream, including *RAS* [10–13], *BRAF* [12, 14], *PIK3CA* [12, 15], contribute to cetuximab resistance. Thus, we assumed that, as the downstream signal pathway of EGFR, the alteration of MAPK pathway might also play a role in cetuximab resistance. In this study, we found that the phosphorylation of MEK/ERK proteins increased after AEP overexpression, and MEK inhibitors U0126 could partially block the resistance induced by AEP. U0126, as an inhibitor of the MAPK signaling pathway, is closely related to various biological processes, such as differentiation, cell growth, autophagy, apoptosis, and stress responses. It makes U0126 play an essential role in various cancers [32].

It has been well reported that AEP may promote cancer progression through diverse pathways, but little is known about the mechanism of AEP-mediated drug resistance. AEP could promote tumor progression by blocking the tumor-suppressive function of P53 [26], activating the PI3K/AKT pathway [33], remodeling of the extracellular matrix (ECM) [34], increasing endothelial permeability [35], or modulating epithelial-to-mesenchymal transition (EMT) [36]. AEP may also be involved in the immune regulation process. In experimental colitis and graft versus host disease (GVHD) mice, the researchers found that programmed cell death ligand 1 (PD-L1) maintains the intrinsic function of Foxp3 by specifically lowering the AEP in lysozyme [37]. AEP is an upstream activator of the cathepsin L-C3-IFN-gamma axis in human CD4(+) T cells and hence an important supporter of human Th1 induction [38]. All these mechanisms may also contribute to drug resistance and further research is needed.

In this study, AEP inhibitors (AEPIs) were used to restore the sensitivity of AEP-overexpressed cells to cetuximab. Another study [39] has shown that AEP-specific small molecule inhibitor can effectively inhibit tumor progression and improved survival in breast cancer transgenic mice. Therefore, AEP may be an effective target for tumor treatment or reversal of drug resistance in the future. Referring to the reported strategy, the following therapeutic methods are proposed for consideration: a combination of medications consisting of signaling pathway inhibitors and AEPI or immunotherapy plus AEPI. Moreover, what triggers the up-regulation of AEP in cancers has not been well known. The concrete signaling pathway regulation of the AEP protein and the activation of AEP are worth exploring and will be helpful for blocking the carcinoma-promoting effect of AEP.

In conclusion, this study suggested that the higher expression of AEP could contribute to the shorter PFS of cetuximab treatment in mCRC. The sensitivity of AEP overexpression colon cancer cells to cetuximab decreased, which can be partially restored by AEP inhibitor. AEP may mediate cetuximab resistance by activating the phosphorylation of EGFR/MEK/ERK signaling pathway.

## Supplementary Information

Below is the link to the electronic supplementary material.

Supplementary file1 (DOCX 110 KB)
