# Supplementary material for: Asparaginyl endopeptidase contributes to cetuximab resistance via MEK/ERK signaling in RAS wide-type metastatic colorectal cancer
Source: Clin Transl Oncol. 2023 Jan 7;25(3):776–85. doi: 10.1007/s12094-022-02986-6 (PMC9941237; doi:10.1007/s12094-022-02986-6)
Supplement: Supplementary file 1 — Supplementary file1 (DOCX 110 KB) [file 12094_2022_2986_MOESM1_ESM.docx]

**Supplement Table 1：Primers used in qPCR**

| LGMN-f | TCGTCCTACGCCTGTTACTA |
| --- | --- |
| LGMN-r | GATCTTCCACGTCCGAATCTT |
| GPR137B-f | GTCTCTCTCTCCATCTGTCTCT |
| GPR137B-r | CCGAGAGGTGTAAAGCAGTATC |
| FAM3A-f | GTCAACGACCTGTTGAAGTTTATT |
| FAM3A-r | CTGAAGAGCTTTCTGGTCTCTT |
| PRNP-f | GGTGGTGTCTCACTCTTTCTTC |
| PRNP-r | CCAGCATCTCAGGTCTACTCTA |
| FSTL3-f | GAGGAAGAGAACTTCGTGTGAG |
| FSTL3-r | GGAGTGTCCGTGGCATATAAA |
| ITGA5-f | GGTGGACCAGGAAGCTATTT |
| ITGA5-r | GAACCAGGTTGATCAGGTACTC |
| CSAD-f | CTAGTCATGGAGCCTGAGTTTG |
| CSAD-r | TGACAGCCTTTCGTGGTAATC |
| NR1H2-f | AGAGCGCAAGCGAAAGAA |
| NR1H2-r | TGAGCACGTTGTAGTGGAAG |

**Supplement Table 2. The top 100 upregulated DEGs identified from GSE5851 profile**

| **Gene** | ***P* Value** |  | **Gene** | ***P* Value** |
| --- | --- | --- | --- | --- |
| RCBTB2 | 0.00005 |  | CLIC2 | 0.00373 |
| PLS3 | 0.0001 |  | NRBF2 | 0.00374 |
| SNX24 | 0.00012 |  | AUH | 0.00378 |
| HAMP | 0.00013 |  | TNFRSF10B | 0.0038 |
| FBXL5 | 0.00016 |  | CEBPD | 0.00415 |
| INSIG2 | 0.00016 |  | DNAJB4 | 0.00419 |
| SERINC1 | 0.00019 |  | C16orf62 | 0.00431 |
| CRP | 0.00028 |  | BMI1 | 0.00447 |
| GPR65 | 0.00032 |  | ARHGEF6 | 0.00449 |
| LYST | 0.00034 |  | TIMM22 | 0.00449 |
| PPP2R1B | 0.00037 |  | TXK | 0.0046 |
| OSBPL1A | 0.00038 |  | LMO4 | 0.005 |
| TLR3 | 0.00043 |  | SERPINB2 | 0.00515 |
| IL13RA1 | 0.00046 |  | ITM2A | 0.00532 |
| JAM2 | 0.00048 |  | ZNF22 | 0.00534 |
| ENPP2 | 0.00062 |  | IGFBP2 | 0.00538 |
| ABHD4 | 0.00071 |  | SHTN1 | 0.00574 |
| HTRA1 | 0.00085 |  | TPD52L1 | 0.00583 |
| SOBP | 0.00085 |  | DAAM1 | 0.00607 |
| GAL3ST1 | 0.00087 |  | MSRB2 | 0.00608 |
| PZP | 0.00096 |  | ARL6IP1 | 0.00625 |
| MYOM1 | 0.00098 |  | ATP7B | 0.00651 |
| MYD88 | 0.00128 |  | FAM49A | 0.00664 |
| CREM | 0.0013 |  | LY96 | 0.00678 |
| AP4S1 | 0.00133 |  | CD226 | 0.00681 |
| LGMN | 0.00133 |  | RGS3 | 0.00685 |
| RASSF9 | 0.00135 |  | SLC35C1 | 0.00702 |
| OSTF1 | 0.00136 |  | SAMSN1 | 0.00723 |
| NUAK1 | 0.00148 |  | SLC35A1 | 0.00754 |
| MECR | 0.0015 |  | RAB14 | 0.00763 |
| RNF11 | 0.00168 |  | TRIB2 | 0.00764 |
| ATG4A | 0.00169 |  | CCDC88A | 0.0077 |
| GNB5 | 0.00169 |  | AKAP12 | 0.00777 |
| ITM2B | 0.0017 |  | BBS10 | 0.00777 |
| PLA2G2A | 0.00173 |  | ITGA1 | 0.00783 |
| RAP2C | 0.00187 |  | ENDOG | 0.00794 |
| LAMP2 | 0.00188 |  | SLC22A18 | 0.0082 |
| IL15RA | 0.00195 |  | FSCN1 | 0.00834 |
| SOCS2 | 0.00225 |  | GNA14 | 0.00844 |
| MEIS3P1 | 0.00228 |  | COQ4 | 0.00885 |
| TNFRSF1A | 0.00245 |  | TLR4 | 0.00899 |
| KDSR | 0.00254 |  | CCDC28A | 0.00923 |
| TRIM8 | 0.00284 |  | ZC3H14 | 0.00934 |
| ZEB2 | 0.003 |  | PLA2G4C | 0.00947 |
| NRP1 | 0.00304 |  | INPP1 | 0.00956 |
| CDK9 | 0.00315 |  | ATG2B | 0.01023 |
| RHOBTB3 | 0.00337 |  | UBE2L6 | 0.01039 |
| HMOX1 | 0.00361 |  | IGSF6 | 0.01048 |
| DSE | 0.00365 |  | MS4A4A | 0.01048 |
| GADD45B | 0.00372 |  | NUCB2 | 0.01075 |
